# Artificial Intelligence in Recurrent Pregnancy Loss: Current Evidence, Limitations, and Future Directions

**DOI:** 10.3390/jcm15020686

**Published:** 2026-01-14

**Authors:** Athanasios Zikopoulos, Efthalia Moustakli, Anastasios Potiris, Konstantinos Louis, Ioannis Arkoulis, Aikaterini Lydia Vogiatzoglou, Maria Tzeli, Nikolaos Kathopoulis, Panagiotis Christopoulos, Nikolaos Thomakos, Ekaterini Domali, Sofoklis Stavros

**Affiliations:** 1 Torbay and South Devon NHS Foundation Trust, Lowes Brg, Torquay TQ2 7AA, UK; thanzik92@gmail.com; 2 Human Computer Interaction Laboratory, Department of Informatics and Telecommunications, University of Ioannina, Kostakioi, 47150 Arta, Greece; ef.moustakli@uoi.gr; 3 Third Department of Obstetrics and Gynecology, University General Hospital “ATTIKON”, Medical School, National and Kapodistrian University of Athens, 12462 Athens, Greece; apotiris@med.uoa.gr (A.P.); garkoylis@hotmail.com (I.A.); vogiatzoglou.lydia@gmail.com (A.L.V.); 4 Department of Midwifery, Faculty of Health and Caring Sciences, University of West Attica, 12243 Athens, Greece; mtzeli@uniwa.gr; 5 First Department of Obstetrics and Gynecology, Alexandra Hospital, Medical School, National and Kapodistrian University of Athens, 11528 Athens, Greece; nickatho@gmail.com (N.K.); nthomakos@med.uoa.gr (N.T.); kdomali@yahoo.fr (E.D.); 6 Second Department of Obstetrics and Gynecology, “Aretaieion” Hospital, Medical School, National and Kapodistrian University of Athens, 11528 Athens, Greece; panchrist@med.uoa.gr

**Keywords:** endometrial microenvironment, immunologic signatures, molecular phenotyping, precision diagnostics, multi-omics profiling, explainable algorithms

## Abstract

Background: Despite significant advances in genetics, immunology, and endometrial research, the underlying cause of nearly half of recurrent pregnancy loss (RPL) cases remains unknown. This highlights the limitations of conventional diagnostic approaches and underscores the need for methods that can detect complex, subtle biological patterns. Objectives: To summarize and critically assess how artificial intelligence (AI) is changing our knowledge of, ability to predict, and future therapeutic management of RPL, with a focus on machine learning (ML) approaches that identify latent biological pathways and multifactorial contributors to pregnancy loss. Methods: This narrative review summarizes contemporary research on AI applications in reproductive medicine. Research using imaging, proteomic, genomic, clinical, and multi-omics information to create predictive or mechanistic models associated with RPL provided evidence. Results: AI-based approaches are increasingly demonstrating the ability to detect complex interactions among environmental, immunological, biochemical, and genetic factors associated with RPL. ML and deep learning (DL) models enhance prognostic accuracy, identify novel candidate biomarkers, and provide insights into the systemic and molecular mechanisms underlying pregnancy loss. Integrating heterogeneous data through AI supports the development of personalized reproductive profiles and can improve prediction and counseling. Conclusions: AI has the potential to improve both personalized prediction and mechanistic understanding of RPL. However, clinical translation is currently hampered by a number of important issues, including small and diverse datasets, conflicting diagnostic definitions, limited external validation, and a lack of prospective clinical trials. To responsibly integrate AI tools into reproductive care, these limitations must be addressed.

## 1. Introduction

Recurrent pregnancy loss (RPL), which affects 1–5% of couples attempting to conceive, is one of the most difficult and upsetting disorders in reproductive medicine. RPL is commonly described as two or more repeated pregnancy losses before fetal viability [1,2]. In addition to persistent issues with diagnosis and treatment, RPL has substantial psychological effects. The complexity of RPL is evident, as major advances in immunology, endocrinology, and reproductive genetics have still left nearly half of the cases unexplained [3].

Conventional research has significantly refined our knowledge of chromosomal abnormalities, uterine defects, antiphospholipid syndrome, endocrine disorders, and thrombophilia. However, such approaches frequently fail to account for idiopathic cases [4]. According to available data, RPL may result from several interconnected mechanisms, including immunological dysregulation, decreased endometrial receptivity, genetic predisposition, changes in the microbiome, and environmental variables. This disjointed viewpoint is indicative of a more general methodological problem with traditional biostatistical techniques, which frequently fall short of capturing the complex interplay of biological processes [5,6,7]. From high-resolution imaging and lifestyle indicators to genetic and proteomic profiles, modern reproductive healthcare generates enormous quantities of unused data. Through the integration of these many datasets, AI presents a potentially valuable analytical framework that facilitates the identification of latent patterns and supports hypothesis generation, although its ability to improve diagnostic accuracy in RPL remains largely unvalidated [8,9].

ML and DL methods have previously demonstrated utility in reproductive medicine, particularly in assessing endometrial receptivity, analyzing sperm morphology, and supporting embryo selection. Early AI-driven studies on pregnancy loss have identified promising predictive patterns in laboratory, genetic, and clinical datasets, as well as tissue-level alterations detectable through imaging and histopathology [10,11]. AI may support the generation of composite biomarkers, defined as multidimensional signatures that surpass the limitations inherent to single-factor analyses by integrating clinical, immunological, and genetic datasets. Explainable AI techniques can offer preliminary insights into candidate biological associations related to miscarriage by clarifying feature significance, although such findings should be interpreted as exploratory rather than mechanistically conclusive [12]. An essential research objective is turning statistical correlations into biologically meaningful hypotheses, a process that has not yet translated into targeted treatments or routine clinical application in RPL [13,14].

Importantly, AI-based approaches in recurrent pregnancy loss remain largely at the exploratory and research stage, with limited prospective validation and no established role in routine clinical decision-making. Clinical confidence could be impeded by small sample sizes, demographic bias, and the “black-box” character of some algorithms unless models are reproducible and transparent. The only way to ensure that AI will be developed and applied in an ethical manner involves interdisciplinary collaboration among physicians, data scientists, and ethicists [15]. This narrative review discusses the current evidence, limitations, and methodological challenges of applying AI to the understanding and prediction of RPL. We discuss the ethical and translational challenges toward clinical adoption, present new biological systems identified using AI, and describe existing applications in reproductive medicine. AI may contribute to future advances in precision reproductive research if methodological, biological, and ethical challenges are adequately addressed, rendering complex data into usable knowledge.

## 2. Materials and Methods

This narrative review is based on a focused but non-systematic search of the scientific literature. Relevant publications were identified through PubMed, Scopus, and Google Scholar up to November 2025 using combinations of the terms “recurrent pregnancy loss,” “miscarriage,” “artificial intelligence,” “machine learning,” “deep learning,” “endometrial receptivity,” “multi-omics,” and “biomarkers.” Priority was given to peer-reviewed clinical, translational, and methodological studies published within the last decade, particularly those addressing reproductive medicine or pregnancy loss.

Studies were included if they applied AI or advanced computational approaches to reproductive biology, implantation, miscarriage prediction, or related mechanistic domains. Evidence quality was assessed informally based on study design, cohort size, validation strategy, and relevance to RPL. Formal systematic review methods, predefined inclusion criteria, and risk-of-bias scoring were not employed, in keeping with the narrative nature of this review.

## 3. The Landscape of AI in Reproductive Medicine

The field of reproductive medicine is currently at the forefront of AI’s therapeutic applications. The intricateness of human reproduction, from gamete interaction to hormone modulation, development of the embryo, and receptivity of the uterus, facilitates data-driven discovery [9]. Over the past ten years, AI applications have progressed from experimental prototypes to widely utilized decision-support systems, improving workflow efficiency, prediction, and diagnostic accuracy in fertility medicine [16]. The specific areas where AI is significantly advancing reproductive practice are thoroughly examined in the sections that follow.

### 3.1. AI in ART and Embryo Selection

The most advanced applications of AI in reproductive medicine currently lie within assisted reproductive technologies (ART). Embryo selection, typically guided by embryologists’ morphological grading, plays a critical role in the success of in vitro fertilization (IVF). Developmental trajectories, blastomere symmetry, and cleavage timing are examples of dynamic morphokinetic features that are currently quantified using AI-based image-analysis algorithms [17,18,19].

In retrospective validation studies, the accuracy of implantation predictions made by convolutional neural networks (CNNs) trained on time-lapse images has been on par with, and in some cases exceeded, that of experienced embryologists. Nevertheless, there is currently insufficient clinical data to demonstrate steady increases in live birth rates, and the evidence is restricted to retrospective or single-center datasets [20].

To investigate biological markers of developmental potential, research-stage AI models are starting to incorporate genomic, transcriptomic, and metabolomic data from embryos or follicular fluid [21,22]. Although these multimodal techniques are still in the exploratory stage, they have the potential to improve embryo-transfer procedures, increase ART efficiency, and reduce the need for multiple transfers. It is crucial to remember that there is still uncertainty regarding the clinical benefits of these supplementary tools, as numerous studies have produced conflicting or ambiguous findings [23].

### 3.2. AI in Endometrial Receptivity and Implantation Dynamics

Endometrial receptivity and embryo quality must coincide for successful implantation. This receptive phase is difficult to define using traditional indicators like sonographic thickness or histologic dates. The idea that receptivity is a personalized, temporally dynamic state rather than a static interval is being more and more supported by developments in transcriptomics and proteomics [24,25].

Transcriptomic-based receptivity testing, including the Endometrial Receptivity Analysis (ERA) and related AI-enhanced classifiers, has generated substantial interest but remains controversial [26,27]. While some studies suggest improved synchronization of embryo transfer timing, randomized trials and systematic reviews have reported inconsistent or negligible benefits in live birth outcomes. These discrepancies likely reflect biological heterogeneity, methodological variability, and limited reproducibility across study populations [28]. Consequently, ERA and similar AI-driven receptivity tools should be regarded as investigational, particularly in the context of recurrent pregnancy loss, rather than universally applicable clinical solutions.

### 3.3. AI in Implantation and Early Pregnancy Outcome Prediction

Beyond endometrial receptivity, AI has also been applied to predicting implantation success and early pregnancy viability, particularly in the context of assisted reproductive technologies. Multivariate ML models incorporating demographic, hormonal, embryonic, and uterine characteristics frequently outperform traditional regression-based scoring systems [29]. Although most models are still constrained by sample size and lack of external validation, ensemble techniques like gradient boosting have produced area-under-the-curve (AUC) values that are close to or higher than 0.85 in several experiments employing carefully selected datasets [30].

Moreover, DL algorithms appear capable of identifying viable versus non-viable early pregnancies by detecting microscopic morphological patterns on ultrasound that are difficult for physicians to perceive. Before being implemented in clinical practice, these first results must undergo prospective validation [31,32]. The range of applications of AI in reproductive medicine is summarized in Table 1, including key domains, data types, and clinical outcomes where ML and DL are increasingly enhancing prediction and diagnostic precision. Collectively, these applications illustrate how methodologies developed in ART and implantation research provide a conceptual and technical foundation for emerging RPL-focused models.

### 3.4. From Prediction to Understanding: Lessons for RPL

Advances in AI-driven prediction in early pregnancy surveillance and ART have informed the study of RPL. While ML analyses of endometrial transcriptomics have revealed possible biomarkers of receptivity, models trained on embryo development trajectories have found crucial temporal thresholds for implantation success [35,36]. Utilizing analogous computational frameworks for RPL may facilitate the identification of subtle factors contributing to loss that are concealed by conventional methodologies, such as immune dysregulation, anomalous placental signaling, or interactions among maternal genetics, the microbiome, and environmental exposures [7].

**Figure 1:**
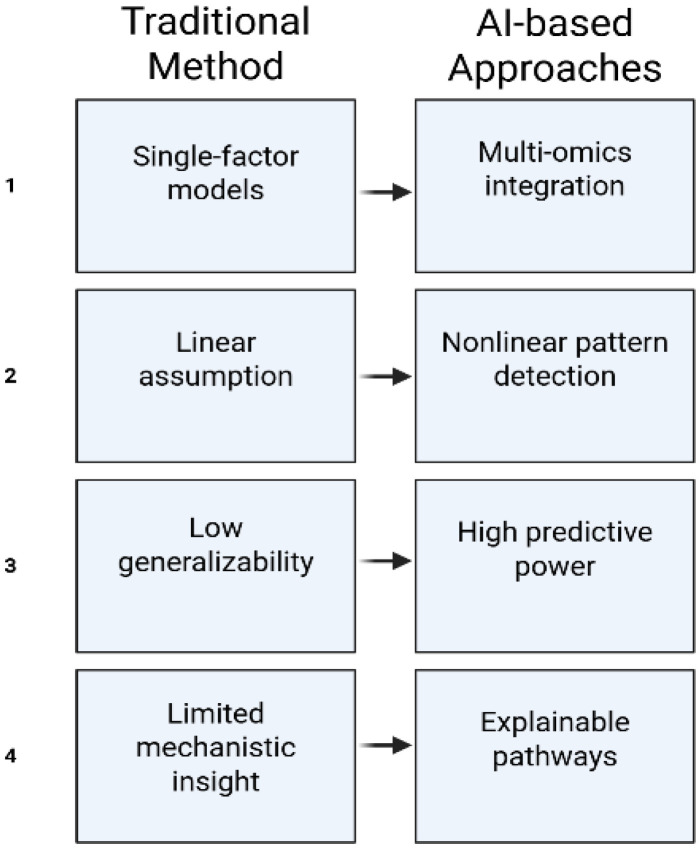
Conceptual comparison between traditional statistical approaches and AI-based methods in reproductive medicine. AI models differ in their ability to integrate heterogeneous data, capture nonlinear relationships, and generate systems-level biological insights, while also introducing challenges related to interpretability, validation, and generalizability.

Substantive progress will necessitate multicenter collaboration supported by harmonized practices, strong data-governance structures, and mutually agreed-upon analytical standards. By using this integrated approach to RPL, it is possible to go beyond prediction toward true mechanistic insight, which could eventually change how RPL is identified and managed [39] (Figure 1). Importantly, most AI applications discussed in reproductive medicine remain at the research or proof-of-concept stage. While certain tools, such as embryo selection algorithms, have undergone retrospective and limited prospective validation, the majority of AI-driven approaches in endometrial receptivity assessment, multi-omics integration, and RPL risk prediction lack robust external validation or regulatory approval. Distinguishing between exploratory computational models and clinically validated decision-support systems is essential to avoid premature clinical translation and unrealistic expectations.

## 4. AI in RPL: Current Evidence

The absence of consistent diagnostic definitions across research is a major obstacle to using AI in RPL [40,41]. Significant heterogeneity in datasets is caused by variations in criteria, such as whether RPL is classified after two or three losses, variations in gestational age requirements, and uneven application of international recommendations. This discrepancy reduces the generalizability of AI-driven conclusions, restricts model comparability, and makes training and validation more difficult [42,43].

In reproductive medicine, RPL is still one of the most complicated and poorly understood conditions. Its investigation is further constrained by the breadth and variability of the data, together with the nonlinear interactions that occur among genetic, immunologic, and environmental factors [44,45]. By integrating diverse datasets to uncover latent patterns, AI offers an exploratory analytical approach for studying such complexity. Recent studies in genomics, immunology, endometrial biology, and clinical prediction have indicated that AI may contribute to exploratory insights into RPL, although its direct application to this disorder remains at an early research stage [46,47].

### 4.1. Genomic and Epigenomic Insights

Genetic factors represent a significant area of research in RPL and include chromosomal abnormalities, single-gene mutations, and polygenic susceptibilities. However, conventional tests explain only a small percentage of recurrent instances [48]. Large genomic data generated through next generation sequencing (NGS) are ideally suited for AI-driven research. Supervised learning algorithms have demonstrated the potential to prioritize miscarriage-associated variants more effectively than manual annotation methods [49]. Integrated analysis of genomic, transcriptomic, and proteomic data using methods such as random forests and gradient boosting points toward potential genes linked to immunological tolerance, placental development, and implantation failure [50].

AI methods have additionally been employed in epigenetic profiling, encompassing DNA methylation and histone-modification patterns together with analyses of sequence variation [51,52]. These investigations have identified aberrant expression within pathways associated with immune modulation, trophoblast invasion, and angiogenesis. Such findings indicate that tailored molecular risk profiles may complement, rather than substitute for, conventional binary classifications, thereby permitting the development of more sophisticated etiologic models of RPL [53,54].

### 4.2. Immunologic and Inflammatory Networks

Immune dysregulation has long been recognized as a possible contributor to recurrent miscarriage, yet it remains difficult to describe clearly. Maternal immunological tolerance and defense must be in a well-regulated balance for successful implantation and placentation [44,55]. Early applications of ML have begun to capture this complexity. In exploratory studies, unsupervised clustering algorithms have revealed potential patient subgroups defined by cytokine patterns and immune-cell composition rather than clinical criteria alone. Neural network–based models combining cytokine profiles, T-cell subset ratios, and NK-cell functional measures have shown favorable predictive accuracy in small, retrospective datasets, although broader validation is still needed [56]. Although some cited studies originate from non-reproductive fields such as cancer metabolomics, they are included here to illustrate transferable AI methodologies for analyzing high-dimensional immune and biomarker data that are increasingly applicable to RPL research.

DL methods have suggested candidate gene-expression networks potentially involved in trophoblast–immune interactions and enabled preliminary modeling of the maternal–fetal interface by incorporating single-cell transcriptomic data [57,58]. Collectively, the emerging evidence indicates that recurrent loss may be driven less by obvious immunologic abnormalities and more by subtle shifts in immune signaling, which AI may be capable of characterizing more systematically as larger datasets become available.

### 4.3. Endometrial Receptivity and Microenvironment Analysis

Endometrial function is closely linked to the success of implantation and is regulated by a complex interplay of hormonal, immunologic, and structural signals. Modeling this temporally dynamic system has long been challenging [59,60]. AI-based analyses of transcriptomic and proteomic datasets have identified receptive and non-receptive endometrial profiles, particularly in studies of ART and implantation failure, with emerging exploratory applications in women with unexplained losses. Microscopic alterations in glandular shape, stromal edema, and vascular density that are undetectable by human inspection can also be unveiled through DL interpretation of histological images [61,62].

Integrating these molecular and histologic findings with systemic factors such as hormone levels and immune profiles enables the development of multimodal AI models capable of generating individualized implantation profiles [63]. In the context of RPL, such approaches hold the potential to support personalized diagnostics and, in the future, may help guide the timing of embryo transfer or targeted therapeutic interventions, although these applications remain investigational [64].

### 4.4. Predictive Modeling and Clinical Decision Support

AI-based predictive methods for RPL risk and recurrence have emerged in recent years, primarily within exploratory and retrospective research settings. Compared to standard logistic regression in exploratory datasets, machine learning models are better able to identify nonlinear relationships between variables such as maternal age, BMI, hormone levels, and thrombophilia indicators by integrating demographic, biochemical, and obstetric variables [65]. Ensemble methods, including XGBoost and random forest, have reported AUC values approaching or exceeding 0.85 in selected retrospective cohorts; however, these findings are often derived from single-center datasets, and external validation and broader generalizability [66]. Although some cited performance benchmarks originate from non-reproductive fields such as oncology, they are referenced here to illustrate transferable machine-learning evaluation frameworks applicable to RPL risk prediction. This raises concerns regarding overfitting and the robustness of model performance across diverse populations.

In addition to improving interpretability, the use of explainable AI (XAI) tools to identify significant predictors may help highlight biologically relevant factors and pathways that are pertinent to clinical decision-making. Such approaches can support clinician understanding by clarifying the relative contribution of features such as hormonal, immunological, or endometrial parameters. Nevertheless, XAI outputs should be interpreted cautiously, as feature-attribution methods may be unstable in small or highly correlated datasets and do not imply causality.

Integrated into electronic health record systems, such tools could ultimately provide real-time risk stratification and personalized management [67]. However, the majority of AI-based predictive models for RPL remain in early developmental stages and require prospective validation, standardized outcome definitions, and demonstration of clinical utility before routine implementation in clinical practice.

### 4.5. Multi-Omics Integration and Systems-Level Understanding

One of the most promising directions in AI-driven RPL research is its capacity to integrate heterogeneous data types into cohesive, biologically informed frameworks [68]. Approaches including autoencoders, graph neural networks, and Bayesian inference can contribute to identifying potential “tipping point” for pregnancy loss by modeling interactions between gene expression, microbial profiles, and underlying immune or hormonal pathways [69]. As multi-omics resources expand and analytical tools advance, such integrated approaches may support future hypotheses for customized, biologically informed treatment strategies, including hormone-based therapies, microbiome-directed interventions, immunomodulation, and anticoagulation [70]. Table 2 summarizes representative applications of AI across the molecular, immunological, and clinical domains of RPL discussed in this section. Most AI applications in RPL remain exploratory, hypothesis-generating, and derived from small or heterogeneous datasets, with limited external or prospective validation.

### 4.6. Limitations and Future Challenges

AI has great potential to improve human comprehension of RPL, but its application is still difficult. Due to the condition’s relative rarity and clinical heterogeneity, datasets are usually tiny, fragmented, and institution-specific [3]. The development of reliable algorithms that function consistently across populations is hampered by this unpredictability, which also restricts the generalizability of models [79].

In addition to imbalanced datasets and biased clinical or demographic samples, predictive accuracy can also be impacted by variations in reference standards, preprocessing procedures, or outcome definitions between studies [80]. Transparency and interpretability are particularly important when it comes to pregnancy loss, as algorithmic results may have a direct impact on patient decision-making and professional counseling [81].

Addressing these limitations would require strong interdisciplinary governance encompassing doctors, data scientists, and ethicists, as well as standardized data standards, explicit reporting guidelines, and thorough validation procedures [82,83]. The prediction ability of existing AI models may not generalize across a variety of populations in the absence of strong external validation across independent and multi-center cohorts [84]. A recurrent failure mode of AI models in RPL research is overfitting, particularly in studies using high-dimensional genomic or multi-omics data derived from small cohorts. Models may demonstrate excellent internal performance while failing external validation due to population heterogeneity, batch effects, or subtle differences in clinical definitions. Inadequate generalizability across ethnic, geographic, and healthcare settings further limits real-world applicability. These limitations underscore the necessity of multicenter datasets, harmonized definitions, and independent validation before clinical implementation.

## 5. Challenges, Limitations, and Ethical Considerations

Although AI has great potential to advance our understanding of RPL, its therapeutic utility depends on the resolving important methodological, technical, and ethical issues [85,86]. Table 3 summarizes the ethical, governance, and methodological barriers to responsible AI integration in RPL research and clinical practice, along with representative mitigation strategies.

### 5.1. Data Availability, Heterogeneity, and Quality

While data constitute the essential substrate for any AI model, RPL research is constrained by ongoing issues of heterogeneity, fragmentation, and insufficient access to comprehensive datasets [94,95]. Unlike cardiology or oncology, fields where large, standardized repositories exist, RPL datasets are usually small, single-center, and variably defined. Cross-study synthesis is complicated by variability in diagnostic definitions, spanning differences in gestational age criteria and in the definitions applied to pregnancy loss [96]. This variability limits the model’s generalizability and increases the risk of overfitting, producing algorithms that achieve high training accuracy but lack dependable real-world performance [97].

### 5.2. Bias, Representativeness, and Interpretability

Predictions are only as unbiased as the data they stem from. Health disparities may be perpetuated because reproductive health records often overrepresent specific ethnic or socioeconomic groups and underrepresent environmental and psychological factors. Inconsistent data labeling and unequal sample sizes further contribute to bias [80,98].

Mitigating these concerns requires continuous bias evaluations, stringent verification of data provenance, and the intentional integration of diverse groups throughout model development [99]. Clinicians can identify possibly inaccurate model outputs and comprehend their underlying reasons with the use of algorithmic explainability tools like feature-importance mapping and Shapley additive explanations (SHAP). By increasing transparency, these approaches can enhance clinical trust and support more informed decision-making.

However, explainability methods also have important limitations. Feature-attribution outputs may be unstable in small datasets or in the presence of highly correlated variables and should not be interpreted as evidence of causal relationships. Consequently, explainable AI should be viewed as a complementary tool that supports, rather than replaces, clinical judgment and biological reasoning. Maintaining long-lasting collaborations between data scientists and physicians is essential to guaranteeing that computational goals continue to represent therapeutically and physiologically relevant requirements [100,101].

### 5.3. Reproducibility, Validation, and Governance

Specific examples demonstrate the practical application of these governance and ethical principle. For instance, explainability tools such as SHAP can enhance transparent clinical decision-making by assisting physicians in comprehending why a model classifies a particular cytokine pattern as high-risk [102]. However, such tools should be interpreted cautiously and within appropriate clinical context, as explainability does not eliminate the need for validation. Similarly, federated learning protects patient privacy while increasing dataset diversity by allowing institutions to train AI models on ultrasound or early-pregnancy data without sharing sensitive patient data. Responsible AI integration in RPL research requires distinguishing between model-level governance concerns such as auditability, bias monitoring, and performance drift and patient-level ethical considerations including consent, privacy, and emotional sensitivity [103].

Given that a considerable share of existing RPL studies employ retrospective designs, lack external validation, or do not meet accepted reporting requirements, achieving reproducibility continues to be a significant obstacle in biological [104]. Improved alignment with contemporary frameworks such as MINIMAR and TRIPOD-AI would substantially enhance methodological transparency, facilitate cross-study comparability, and strengthen the credibility of AI-driven findings in reproductive medicine [105].

### 5.4. Ethical and Human Dimensions

AI adoption in reproductive medicine involves unique ethical considerations regarding privacy, consent, and emotional well-being [88]. Since fertility and pregnancy-related information is particularly private, ethical management will need to go beyond legal compliance to include open communication about data use and sharing. Consent procedures should continue to be completely revocable, active, and informed to protect patient sovereignty over reproductive information [106].

Clinical empathy should be enhanced by AI, not replaced. Technology should encourage empathetic communication rather than impersonal care because pregnancy loss is frequently accompanied by grief and uncertainty [107]. When applied responsibly, AI can serve as a partner in the therapeutic process, enabling clinicians to provide information and support with greater clarity and sensitivity [108]. The extent to which this technology serves as a tool for precision and compassion, rather than perpetuating complexity and inequality, hinges on achieving a balanced integration of computational rigor and human sensitivity [109].

## 6. Future Perspectives and Clinical Translation

AI provides potential opportunities to deepen biological understanding and may, in the future, contribute to patient care in reproductive medicine. Insights gained from early clinical applications, such as embryo selection, endometrial function analysis, and miscarriage prediction, have formed the foundation for a second wave of translational research [9,110].

Future progress will depend on several pivotal directions that will guide AI’s possible evolution from a preliminary research tool toward carefully validated contributions to reproductive diagnostics and personalized care [10,111].

### 6.1. From Prediction to Mechanistic Understanding

The most transformative theoretical potential of AI in RPL extends beyond prediction toward hypothesis-driven mechanistic exploration. Reductionism is a common feature of traditional reproductive science methods, which treat immunologic, genetic, and hormonal factors as distinct entities [112]. AI facilitates the development of integrative frameworks aimed at exploring how environmental, biochemical, and genetic factors may interact to impact reproductive outcomes [113].

The use of network-oriented models and interpretable machine learning techniques may be instrumental in uncovering the biochemical underpinnings of RPL. For instance, graph neural networks can generate experimental testable hypotheses by mapping relationships among immune regulators, trophoblast signaling molecules, and endometrial gene networks [114]. Implementing such systems-level approaches may support a gradual shift RPL research from correlational analysis toward testable causal hypotheses. This direction reflects a broader trend toward AI models based on biological processes and able to generate interpretable scientific insights [115].

### 6.2. Multi-Omics Integration and Precision Reproductive Medicine

AI-enabled multi-omic pipelines can identify high-order biomarkers that transcend any single data type and may ultimately define molecular “signatures” for distinct RPL subgroups [116]. For example, rather than relying solely on specific genetic or hormonal indicators, risk classification may depend on the convergence of immunological expression, uterine microbiota diversity, and systemic metabolic variables [117,118].

Through the use of longitudinal data, such models can follow shifts in biological pathways throughout conception efforts, facilitating individualized and adaptive reproductive interventions. Long-term, individualized research frameworks for evaluating treatment strategies based on a patient’s unique biological architecture, such as immunomodulatory medication, anticoagulation, intrauterine treatments, or microbiome-focused therapies, may eventually be informed by thorough multi-omic analysis [118,119].

However, high costs, complex preprocessing, batch effects, and the small sample sizes typical of RPL cohorts currently limit the use of multi-omics in RPLR, hindering clinical translation and reducing reproducibility [78].

### 6.3. Clinical Implementation and Collaboration

For AI-derived insights to be incorporated into clinical practice, systems must be user-centered and integrate seamlessly into existing workflows. Individualized diagnostic and risk evaluations can benefit from AI systems which integrate genetic, hormonal, imaging, and lifestyle data, as long as the results are understandable and doctors can grasp the underlying reasoning [120,121].

Effective integration with existing EHR infrastructure will also be essential for clinical adoption. In the future, if supported by prospective evidence, AI features integrated into the EHR might automatically identify people at risk, initiate suitable diagnostic procedures, or assess ongoing treatment results [122]. At present, however, prospective clinical trials evaluating AI-based tools specifically in recurrent pregnancy loss remain limited.

### 6.4. Ethical Data Ecosystems and Trust

To properly leverage AI in RPL, large, diverse, and meticulously annotated datasets under stringent ethical norms are necessary [123]. The quality and extent of this data will be decided by national and international data-sharing consortia with an emphasis on reproductive health.

Privacy-preserving approaches, such as federated learning, offer a practical solution by enabling multi-institutional model training while ensuring that sensitive patient data remain local [124,125]. To provide informed consent, protect patient autonomy, and strengthen public trust, ethical and regulatory policy updates must keep pace with these technological advancements. Given its emotional and intimate nature, reproductive health represents a particularly important domain for setting standards in ethical AI governance [126,127].

### 6.5. Education, Interdisciplinary Collaboration, and Workforce Readiness

Close interdisciplinary cooperation is necessary for the appropriate and successful application of AI. To maintain the clinical relevance and ethical soundness of computational techniques, model creation should involve collaboration between clinicians, data scientists, bioinformaticians, and ethicists [128].

Basic AI literacy should be included in reproductive medicine training programs so that doctors can understand model outputs, recognize limitations, and work well with technical experts [9,129]. In a similar vein, reproductive health data scientists need to comprehend the clinical, moral, and psychological aspects of pregnancy and pregnancy loss. To guarantee that AI technologies are both technically sound and in line with patient-centered care, it is crucial to bridge this cultural and disciplinary gap [130,131].

### 6.6. Toward a Human-Centered Future

To ensure that technology strengthens rather than weakens the empathy that is essential to clinician–patient relationships, AI in RPL must continue to be grounded in human values [132]. Prognostic information should be given with both scientific correctness and compassionate understanding because RPL is frequently accompanied by sadness, guilt, and uncertainty [133].

Clinicians can benefit from ethically based AI systems’ ability to process intricate biological facts, provide suggestions backed by science, and tailor treatment plans. In this capacity, AI acts as a link between understanding and prediction, between data analysis and discovery, and between clinical science and empathy [134]. The main future paths, enablers, and ethical issues that will influence the shift in AI in reproductive care toward a mechanistic and human-centered paradigm are listed in Table 4. At present, these future directions should be viewed as research priorities rather than indicators of near-term clinical implementation in RPL.

## 7. Conclusions

In reproductive medicine, RPL is still one of the most complicated and emotionally taxing conditions. The limits of current diagnostic paradigms are highlighted by the fact that over half of cases remain unexplained despite significant advancements in our understanding of endometrial biology, immunological function, and genetic variables. Proteomic and genetic profiles, imaging data, and clinical records are examples of heterogeneous datasets that AI may effectively synthesize to uncover relational patterns and generate mechanistic hypotheses that are frequently overlooked by conventional analytical methods. Unlike many chronic complex diseases, RPL lacks a stable or cumulative disease phenotype, making longitudinal modeling and validation particularly challenging.

Beyond improving prediction, AI may contribute to the generation of novel biological hypotheses. Interpretable models informed by physiological principles can move analyses beyond simple correlations, supporting exploration of how genetic, immunological, and environmental variables may interact to influence reproductive outcomes.

At present, AI-based approaches in recurrent pregnancy loss remain primarily research tools and have not yet demonstrated consistent clinical utility. Clinicians, data scientists, and technological advancements operating within rigorous ethical and methodological frameworks must continue to work together to develop the sector. Integrated with human empathy and robust methodological standards, AI may contribute to future advances in reproductive health, provided that its applications are rigorously validated and ethically governed. This perspective holds that technology can serve as a research collaborator, transforming raw data into insightful knowledge and supporting evidence-informed clinical discussions, rather than replacing established clinical judgment.

## Figures and Tables

**Table 1:** Key applications of artificial intelligence in reproductive medicine. The table summarizes representative AI methods, data sources, and clinical outcomes across major domains of reproductive health, illustrating how computational tools are enhancing precision and prediction from gamete assessment to implantation. Items listed reflect both established and emerging AI applications in reproductive medicine.

Domain/Application	AI Methodology	Data Source/Features Used	Primary Outcomes/Insights
Embryo selection in ART [33]	CNNs Time-lapse ML models	Morphokinetic imaging Blastocyst morphology Developmental timing	Automated embryo ranking Prediction of implantation and live birth
Oocyte and sperm assessment [34]	Image recognition Support Vector Machines (SVMs)	Microscopic images Motility parameters Morphology metrics	Objective gamete quality scoring Fertilization potential prediction
Endometrial receptivity [35,36]	Random forest DL on omics and histology	Transcriptomic/proteomic signatures Histopathology Ultrasound texture	Identification of personalized window of implantation Receptivity biomarkers
Implantation and early pregnancy prediction [37]	Gradient boosting Ensemble learning DL imaging	Hormone levels Embryo quality Endometrial parameters Early ultrasound	Prediction of clinical pregnancy and miscarriage Viability classification
Integration and mechanistic modeling [38]	Multi-omics integration Graph networks Explainable AI	Genomics Proteomics Metabolomics Immune signatures	Systems-level understanding of implantation and RPL mechanisms

**Table 2:** The table summarizes representative AI methods, data sources, and clinical outcomes across major domains of reproductive health, illustrating how computational tools are enhancing precision and prediction from gamete assessment to implantation. Items listed reflect both established and emerging AI applications in reproductive medicine.

Domain/Focus Area	AI Approach/Algorithms	Data Type/Features Used	Key Findings/Outcomes
Genomic and Epigenomic Analysis [71,72,73]	Random Forest, Gradient Boosting, DL	Whole-exome/genome sequencing, methylation profiles, transcriptomics	Identification of novel RPL-associated variants Prioritization of functional genes Epigenetic regulation of trophoblast and immune pathways
Immune Profiling and Cytokine Networks [74,75]	Unsupervised clustering, Neural Networks	Cytokine panels, NK/T-cell ratios, single-cell RNA-seq	Discovery of immune-based RPL subtypes Prediction of miscarriage risk from immune signatures
Endometrial Receptivity and Microenvironment [36,76]	DL, SVMs	Histopathology images, transcriptomic and proteomic data	Detection of subtle endometrial changes linked to implantation failure Personalized receptivity profiling
Clinical Prediction Models [65,77]	Ensemble Learning (XGBoost, Random Forest), Logistic Regression Hybrids	Demographic, hormonal, and obstetric data	Individualized miscarriage risk prediction Explainable models highlighting key predictors
Multi-Omics and Systems-Level Modeling [51,78]	Autoencoders, Graph Neural Networks, Bayesian Models	Integrated genomic, immune, and microbiome data	Identification of molecular phenotypes Holistic understanding of pathophysiological networks

**Table 3:** Key ethical, methodological, and governance barriers to responsible AI integration in recurrent pregnancy loss research and clinical care, along with potential mitigation strategies.

Challenge Area	Specific Issues/Examples	Potential Mitigation Strategies
Data availability and quality [87]	Small, fragmented, single-center datasets Inconsistent RPL definitions (2 vs. 3 losses, gestational age thresholds)	Develop multicenter registries Harmonize diagnostic criteria Adopt federated learning and standardized reporting
Bias and representativeness [88,89]	Overrepresentation of specific ethnic/socioeconomic groups Omission of environmental or psychosocial factors	Ensure dataset diversity Conduct regular bias audits Apply explainable AI [e.g., Shapley additive explanations (SHAP)]
Reproducibility and validation [90,91]	Retrospective studies with no external validation Lack of open datasets	Follow TRIPOD-AI/MINIMAR guidelines Create open-access repositories Require independent validation
Governance and transparency [92,93]	Absence of regulatory oversight Opaque model performance	Establish algorithm auditing frameworks Continuous model updating; regulatory collaboration
Ethical and human dimensions [88]	Privacy, consent, emotional sensitivity of pregnancy loss	Active and revocable consent Privacy-preserving analytics Ensure AI supports without replacing clinical empathy

**Table 4:** This table outlines key future directions in RPL research, their intended impact, and what is needed to achieve them. Each row summarizes one advancement area along with its requirement. Future directions presented here represent emerging trends that remain in various stages of development.

Future Direction	Objective/Potential Impact	Key Enablers/Requirements
Mechanistic AI modeling [135,136]	Move beyond prediction to uncover biological pathways and causal mechanisms underlying RPL	Integration of explainable machine learning, graph neural networks, and systems biology
Multi-omics integration and molecular phenotyping [137,138]	Define molecular RPL subtypes; enable personalized diagnostics and interventions	Coordinated genomic, transcriptomic, proteomic, and microbiome data collection; robust data harmonization
AI-driven clinical decision support [139,140]	Translate computational insights into patient care; improve risk stratification and treatment planning	Integration with EHRs; explainable algorithms; clinician–data scientist co-development
Ethical data ecosystems and global collaboration [141,142]	Ensure transparency, reproducibility, and privacy in AI model development	FAIR data principles; federated learning; international consortia for reproductive data
Education and workforce readiness [143,144]	Equip clinicians and scientists with cross-disciplinary literacy	AI training modules in medical curricula; collaborative professional development
Human-centered AI in care [145,146]	Maintain empathy and trust while using predictive technology	Ethical design; patient empowerment; transparent risk communication

## Data Availability

No new data were created or analyzed in this study.

## Abbreviations

The following abbreviations are used in this manuscript:

RPL	Recurrent Pregnancy Loss
AI	Artificial Intelligence
ML	Machine Learning
DL	Deep Learning
ART	Assisted Reproductive Technologies
IVF	In Vitro Fertilization
CNNs	Convolutional Neural Networks
ERA	Eigensystem Realization Algorithm
AUC	Area Under the Curve
SVMs	Support Vector Machines
NGS	Next Generation Sequencing
